# Supplementary material for: Dietary and Biological Assessment of the Omega-3 Status of Collegiate Athletes: A Cross-Sectional Analysis
Source: PLoS One. 2020 Apr 29;15(4):e0228834. doi: 10.1371/journal.pone.0228834 (PMC7190167; doi:10.1371/journal.pone.0228834)
Supplement: Attachment — Submitted filename: Response to Reviewers 4.3.2020.docx [file pone.0228834.s002.docx]

Dr. Sauers:

Genuine thanks for the additional review of our manuscript, PONE-D-20-02046R1 “Dietary and Biological Assessment of the Omega-3 Status of Collegiate Athletes: A Cross-Sectional Analysis”. We have made the three additional valuable edits recommended by the reviewer, as outlined below. Please let us know if any additional information is required.

We sincerely hope you are doing well during this challenging time.

Sincerely,

Michelle Rockwell and Peter Ritz

| Reviewer #1:  • Please specifically list each Power 5 conference in the area in which it is first introduced to provide specificity to readers that may not know these conferences in a paratheatrical note. | This edit has been made. |
| --- | --- |
| • The authors indicate that participating institutions were assured protection of program identity; however, it is known that some states that only have one Power 5 institution will be identified by default (e.g., University of Utah). This should be listed as a limitation. | This edit has been made. |
| • The authors did a nice job of addressing the concern related to comparing football and non-football athletes in their revisions letter; however, the limitations of making these comparisons should also be listed in the limitations paragraph. | Thank you. This edit has also been made. |
